# Gender Disparities in Out-of-hospital Cardiac Arrests

**DOI:** 10.7759/cureus.3233

**Published:** 2018-08-30

**Authors:** Glenn Goodwin, Dyana Picache, Nicholas Gaeto, Brian J Louie, Tarik Zeid, Paxton P Aung, Sonu Sahni

**Affiliations:** 1 Department of Osteopathic Medicine, Touro College of Osteopathic Medicine, New York, USA; 2 Department of Primary Care, Touro College of Osteopathic Medicine, New York, USA; 3 Student, Touro College of Osteopathic Medicine, New York, USA; 4 Department of Internal Medicine, Brookdale University Hospital Medical Center, New York, USA

**Keywords:** cardiac arrest, gender, prehospital care, ems, health disparities, resuscitation, cardiopulmonary resuscitation, defibrillation, intubation, rosc

## Abstract

Background

Despite advances in resuscitation science and public health, out-of-hospital cardiac arrest (OOHCA) has an average survival rate of only 12% nationwide, compared to 24.8% of patients who suffer from cardiac arrest while in hospital. Additionally, gender is an important element of human health, and there is a clear pattern for gender-specific survivability in cardiac arrest. This study examined differences in presentations, treatment, management, and outcomes.

Aim

The primary focus of this study was to shed light on differences in presentations, treatments, and outcomes between men and women suffering from an out-of-hospital cardiac arrest and the accompanying contributing factors.

Methods

All emergency medical services-related data, including age, date, initial rhythm, chemical interventions (i.e., epinephrine, dextrose), blood glucose levels, defibrillations, endotracheal tube (ETT) attempts, final airway management, achievement of return of spontaneous circulation (ROSC), and the conclusion of the case up to the emergency department, were recorded using a standardized emergency medical services (EMS) charting record by the highest-ranking EMS provider on the ambulance. The reports were retrospectively collected and analyzed.

Conclusion

The study examined demographics, treatments rendered, and outcomes in OOHCA cases that occurred in a major United States (US) city in 2016. Several significant differences in care were noted between men and women. In general, women received less respiratory, chemical, and electrical interventions than men; however, statistically significant differences were only observed in the number of attempts of endotracheal intubations, number of doses of epinephrine per encounter, and number of defibrillations per encounter. In spite of generally receiving less care, women appeared to respond more favorably to cardiac arrest interventions, as demonstrated by higher rates of ROSC. Despite this, women were also found to be eight years older at the time of arrest. Future studies are needed to determine causality in discrepancies between the genders in addition to investigating differences in treatment in other areas of the United States.

## Introduction

Out-of-hospital cardiac arrest (OOHCA) is a life-threatening emergency that affects more than 350,000 Americans a year. Despite advances in resuscitation science and public health, OOHCA has an average survival rate of only 12% nationwide, compared to 24.8% of patients who suffer from cardiac arrest while in hospital [1]. Demographic differences, such as race, socioeconomic status, and gender, have been shown to impact OOHCA survivability [2-3]. Our study attempts to highlight the differences in outcomes of OOHCA between male and female patients through several parameters. Factors such as emergency medical service (EMS) training and capabilities, cultural norms regarding gender and their effect on treatment, and the aggressiveness of cardiac arrest treatment, especially for certain presenting conditions, are all contributing factors to patient outcomes between genders [4-6]. Because these elements are usually contingent on the society and location in which the cardiac arrest took place, varying levels of disparities would be expected to be seen throughout different areas of the world.

A hinderance with researching prehospital care is the significant variation of education, scope of practice, and service delivery between emergency medical services (EMS) organizations, which can change even between adjacent municipalities. Because of this, it is important to highlight some of the aforementioned features of advanced life support (ALS) paramedics in the Miami Fire-Rescue system. Miami Fire-Rescue operates 26 advanced life-support ambulances, staffed by two to three firefighter/paramedics [7]. Miami’s paramedics are trained at an ALS level of care, including the placement of endotracheal tubes (ETT) and supraglottic airways, intravenous (IV) access, administration of various medications, manual defibrillation, cardioversion, transcutaneous pacing, end-tidal carbon dioxide (CO_2_) analysis, and blood glucose monitoring. Practicing under the protocols of the Miami Fire-Rescue department, paramedics exercise clinical judgment to decide which interventions are appropriate to administer [8-9].

This retrospective study examined cardiac arrest episodes that were treated by the City of Miami Fire-Rescue Department during the year 2016. A patient outcome was deemed positive if the patient achieved return of spontaneous circulation (ROSC). Differences in initial rhythm, airway management, rates of ROSC, age of arrest, and deliverance of various interventions, outcomes, and responses to treatment between genders were the parameters investigated in this study.

## Materials and methods

Data collected for this retrospective, observational study were obtained from the City of Miami Fire Department with their expressed written consent. Patient confidentiality was upheld in accordance with Health Insurance Portability and Accountability Act (HIPAA) standards.

Study setting

This study took place in the city of Miami, with an estimated population size of 453,000, during the year 2016. The 2016 self-reported demographics include 75.6% White or Caucasian (including White Hispanic), 71.2% Hispanic or Latino (of any race), 18.8% Black or African-American, 11.2% Non-Hispanic White or Caucasian, 0.9% Asian, and 0.2% Native American or Native Alaskan. The varying percentages are a result of people identifying as more than one race; however, it is worth noting that the majority of the Miami population identified as Hispanic or Latino. The median household income between 2011-2015 was $43,129, with 20% of the population living in poverty [10].

Study population

**Figure 1:**
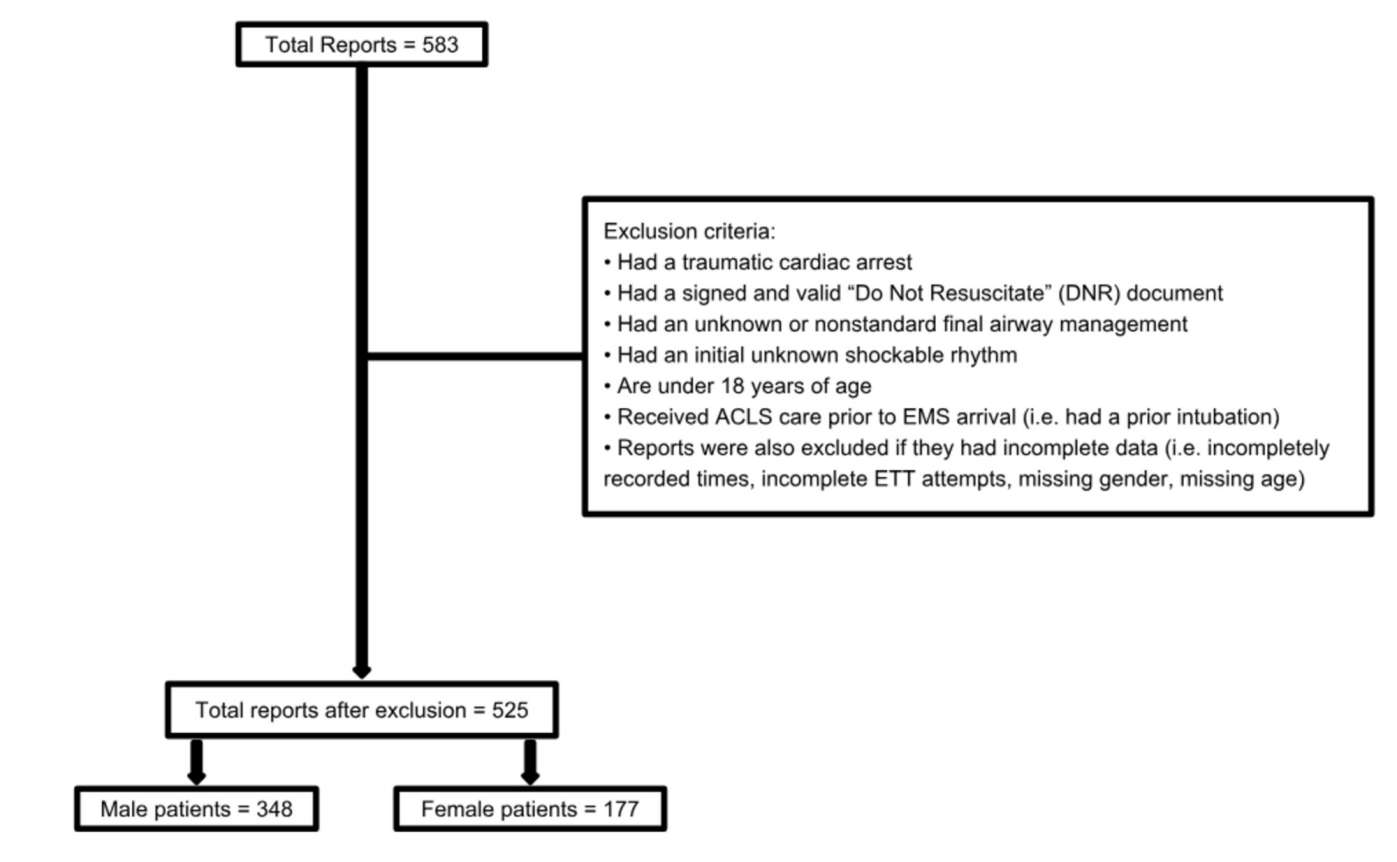
Patient Enrolment ACLS: Advanced Cardiac Life Support

The focus of this study centered around patients suffering out-of-hospital cardiac arrest, treated by City of Miami Fire-Rescue, in the year 2016. The study initially had 583 cardiac arrest reports and following exclusion, 525 patients were included in the final research. The 525 patients included 348 males and 177 females of various ethnicities. Patients ranged from 19 to 100 years old. Exclusion criteria and breakdown is provided in Figure 1.

Data collection

All EMS-related data, including age, date, initial rhythm, chemical interventions (i.e., epinephrine, dextrose), blood glucose levels, defibrillations, endotracheal tube (ETT) attempts, final airway management, achievement of ROSC, and the conclusion of the case up to the emergency department, were recorded using a standardized EMS charting record by the highest-ranking EMS provider on the ambulance. The reports were retrospectively collected and analyzed by the authors of the study.

Data variables

The study had one independent variable: gender. The dependent variables included average patient age, initial rhythm, final airway management, number of ETT attempts, number of rounds of epinephrine, number of defibrillations, dextrose administration (if patient’s blood glucose level was <60 mg/dL), ROSC, and ultimate prehospital outcome. ROSC is defined as a palpable pulse in any vessel for any length of time [11]. ROSC attained at any point and for any duration during the encounter was recorded in the study.

Outcome measure

Discrepancies in care between males (M) and females (F) was the endpoint of interest.

Statistical analysis

Numerical values in this study, such as presenting age, number of doses of epinephrine, number of defibrillations, were analyzed using the student’s unpaired t-test with an alpha level of 0.05. Categorical data, such as initial presenting rhythm, whether or not ETT was attempted and success rates, final airway management, whether or not dextrose was administered, and ultimate prehospital outcomes, were analyzed using the chi-square test of independence with an alpha level of 0.05. All statistics above were examined for differences between the treatment and outcomes of males and females.

## Results

Demographics and clinical presentation

The current study examined differences between males and females in demographics, delivery of care, and clinical outcomes. A total of 525 patients were included in this study and were separated on the basis of gender. There were appreciably more males, 348, as compared with females, 177. As demonstrated in Table 1, women suffering from OOHCA were approximately eight years older than their male counterparts, which is a statistically significant age difference.

**Table 1:** Average Age Females (F) were on average eight years older than males (M) in the data set. An unpaired t-test was used to compare the average ages and the difference was found to be significant (p = 0.0002; confidence interval (CI): -10.64, -3.39). (SD = standard deviation, SEM = standard error of the mean, N = number of patients)

	M	F
MEAN	63.97	71.81
SD	17.58	18.42
SEM	0.94	1.38
N	348	177

**Figure 2:**
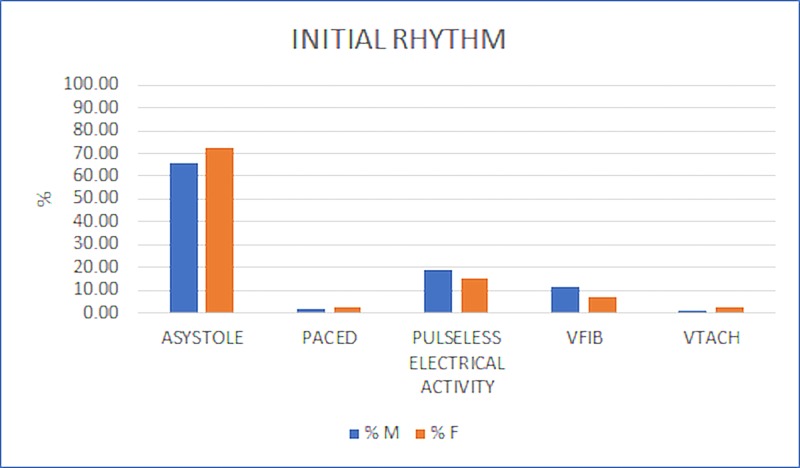
Initial Rhythm The majority of both males (M) and females (F) in the data set presented with asystole as the initial arrest rhythm (68%), followed by pulseless electrical activity (PEA) (18%), ventricular fibrillation (VFIB) (10%), paced rhythm (PACED) (2%), and ventricular tachycardia (VTACH) (2%). A greater percentage of females presented with asystole (M: 65%; F: 72%), VTACH (M: 1.4%; F: 2%), and PACED (M: 1.7%; F: 3%) while a greater percentage of males presented with VFIB (M: 12%; F: 7%) and PEA (M: 19%; F: 15%). A chi-squared test for independence was used to compare the number of subjects with each presenting rhythm. The relationship between initial rhythm and gender was not significant, chi-squared (4, N = 525) = 5.16, p = 0.27.

Women were marginally more likely to initially present in asystole and ventricular tachycardia (VTACH) while men presented more frequently in pulseless electrical activity (PEA) and ventricular fibrillation (VFIB) (Figure 2). The higher rate of non-shockable rhythms seen in women is consistent with similar such studies [3].

Deliverance of care

**Figure 3:**
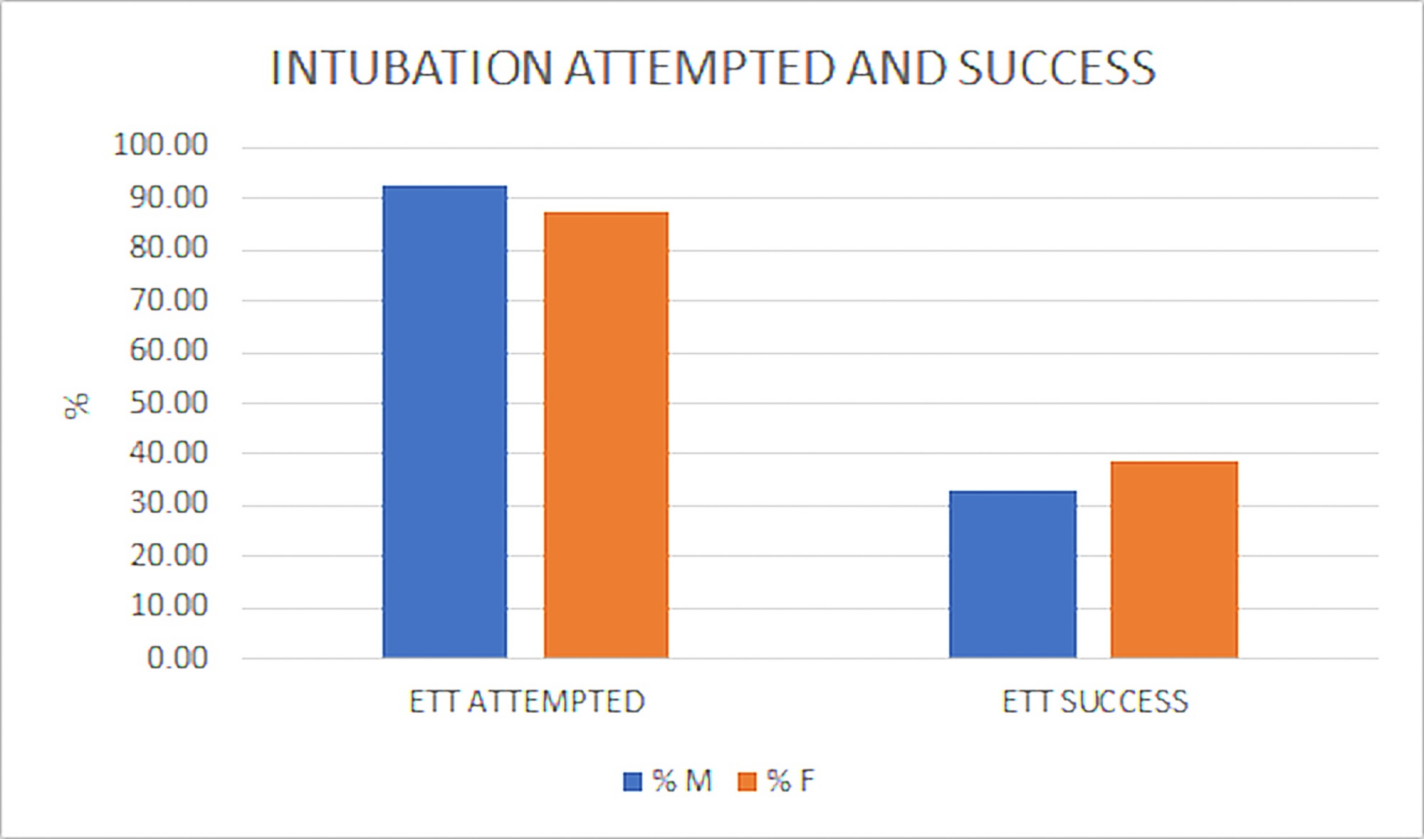
Intubation Attempted and Success Ninety-two percent of all male (M) and 87% of all female (F) arrest cases received an ETT attempt. A chi-squared test of independence was used to compare the total number of ETT attempts by gender and the difference was significant, chi-squared (1, N = 525) = 3.96, p = 0.04. Although female subjects were significantly less likely to receive an ETT attempt, there were no significant differences between the success rates of the intubation (32% successful male ETT, 38% successful female ETT, chi-squared (1, N = 478) = 1.60, p = 0.20).

Regarding treatments, females had significantly fewer ETT attempts than males. Despite receiving fewer ETT attempts, the ETT success rate was higher in women, although not statistically significant (Figure 3).

**Figure 4:**
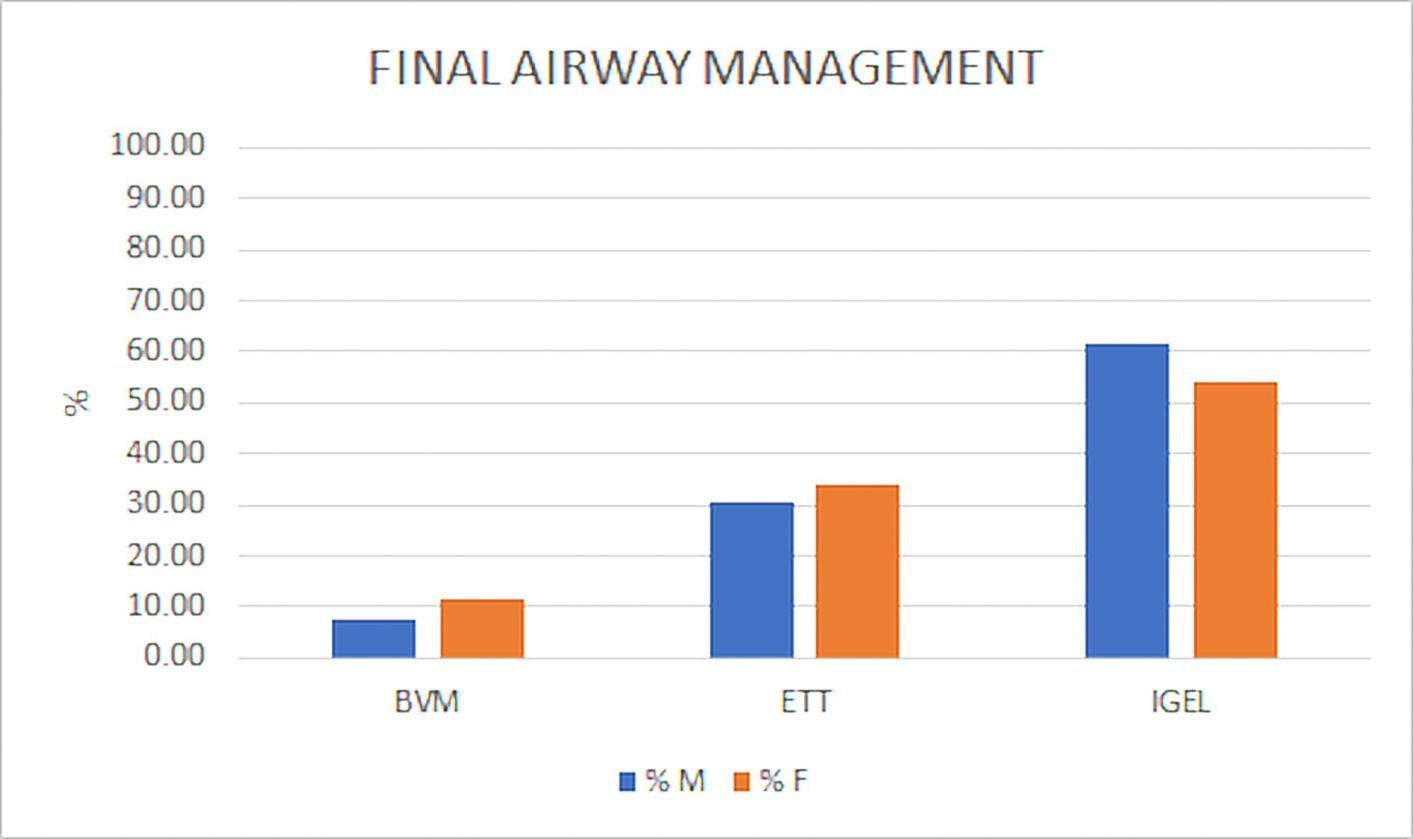
Final Airway Management Most subjects received a supraglottic airway (IGEL) as final airway management. A minority of subjects were ventilated with no advanced airway, using a bag valve mask (BVM). There was no significant difference between final airway management in males (M) versus females (F), chi-squared (2, N = 525) = 3.72, p = 0.15.

Despite the difference in ETT attempts, the final airway management between genders did not vary significantly (Figure 4).

**Figure 5:**
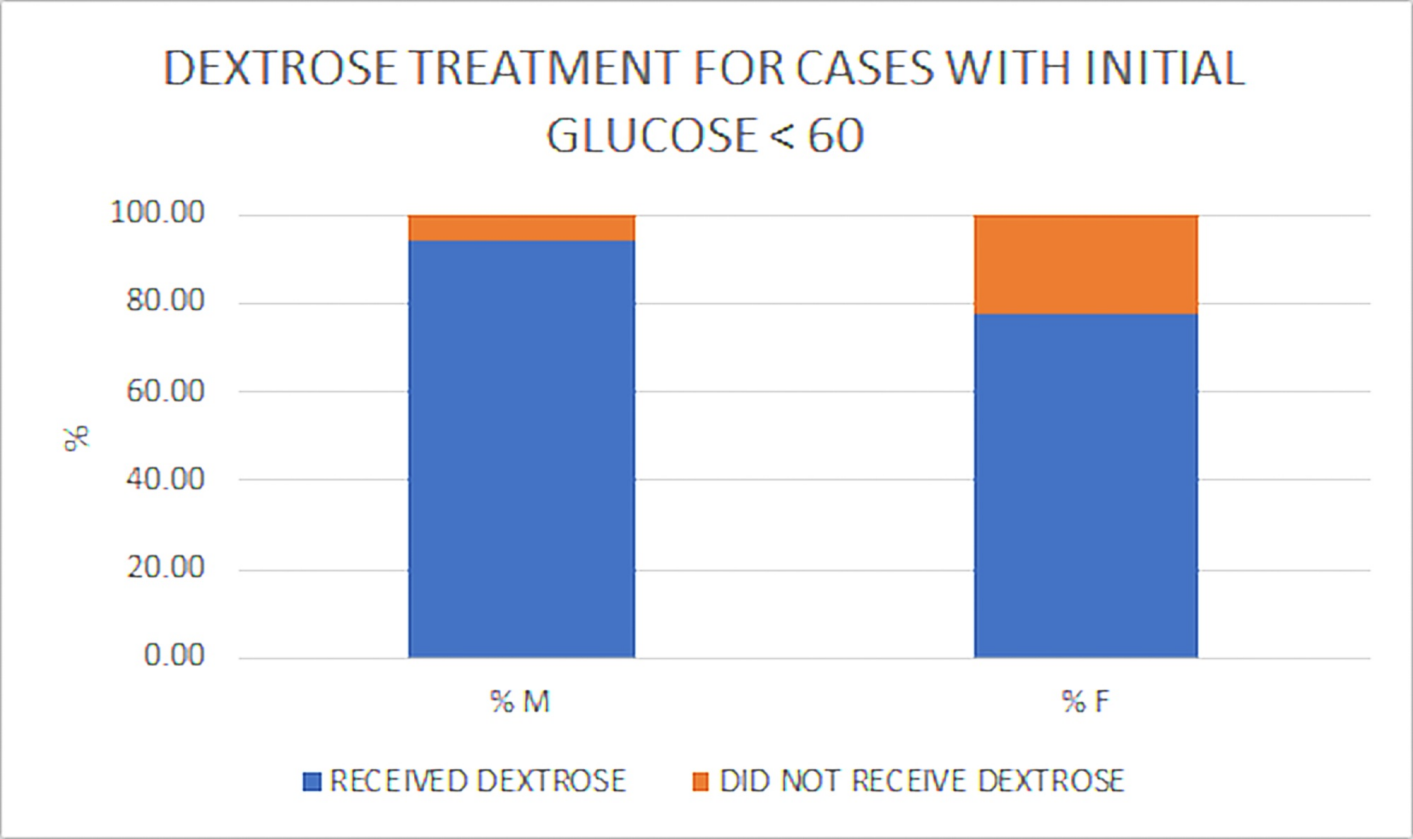
Dextrose Administration Dextrose administration. This subset of data included cases with an initial recorded glucose < 60 mg/dL (i.e. 59 and below). Approximately 16% more males (M) with blood glucose < 60 mg/dL received dextrose as an intervention than females (F) with similar presentation. (Received dextrose M: 93.94%; F: 77.78%). However, this difference was not significant, chi-squared (1, N = 51) = 2.93, p = 0.08.

Other observed differences in care included the number of epinephrine doses (Table 2), number of defibrillations (Table 3), and administration of dextrose (Figure 5), all interventions in which males received more treatments. However, significance was only observed for the number of epinephrine doses and number of defibrillations.

**Table 2:** Epinephrine Doses Per Encounter Epinephrine (EPI) doses per encounter. This analysis includes all subjects from the study due to the fact that each subject was eligible to receive at least one dose of epinephrine. Males received significantly more doses of epinephrine per encounter than women, p = 0.03; CI: 0.02, 0.79. (SD = standard deviation, SEM = standard error of the mean, N = number of patients)

	NUMBER OF MALE EPI DOSES	NUMBER OF FEMALE EPI DOSES
MEAN	4.39	3.99
SD	2.09	2.19
SEM	0.11	0.16
N	348	177

**Table 3:** Number of Defibrillations Per Encounter Number of defibrillations (DEFIBS) per encounter. This subset of data included only subjects that presented with an initial shockable rhythm of either VTACH or VFIB. Males received significantly more defibrillations per encounter than women, p = 0.02; CI: 0.15, 2.49. (SD = standard deviation, SEM = standard error of the mean, N = number of patients)

	NUMBER OF MALE DEFIBS	NUMBER OF FEMALE DEFIBS
MEAN	3.26	1.94
SD	2.23	1.48
SEM	0.33	0.36
N	46	17

Outcomes and response to treatment

It is worth noting that in addition to differences in initial patient presentation and treatment, the overall rates of ROSC (Table 4) and the prehospital conclusions of the encounters (Figure 6) also differed between genders; although they were not statistically significant.

**Table 4:** Rates of Return of Spontaneous Circulation Approximately 7% more females than males achieved ROSC. A chi-squared test of independence showed no significant difference in ROSC in males (M) and females (F), chi-squared (1, N = 525) = 2.90, p = 0.08.

	#M	#F	TOTAL	%M	%F	% TOTAL
ROSC	106	67	173	30	37	33
NO ROSC	242	110	352	69	62	67
TOTAL	348	177	525			

**Figure 6:**
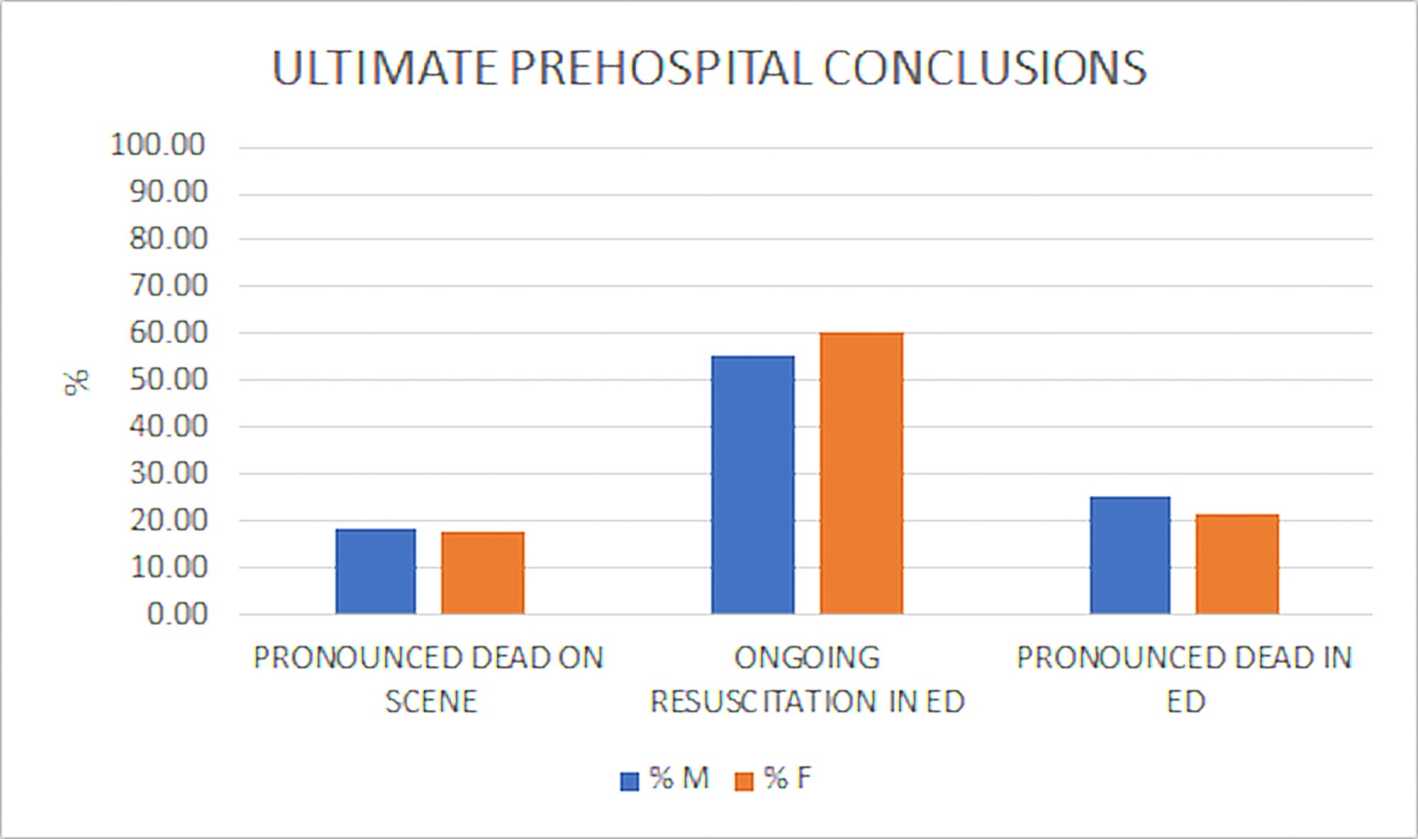
Ultimate Prehospital Conclusions The majority of cases resulted with ongoing resuscitation in the Emergency Department (ED). Percentages were similar between males (M) and females (F) for all outcomes. Pronounced dead on scene, M: 18%; F: 18% Pronounced dead in ED, M: 25%; F: 21% Ongoing resuscitation in ED, M: 55%; F: 60% A chi-squared test of independence showed no significant difference in ultimate prehospital conclusion in males and females, chi-squared (2, N = 525) = 1.29, p =0.52.

## Discussion

Gender is an important element of human health, and there is a clear pattern for sex-specific survivability in cardiac arrests. While this is not a new finding, the underlying factors and implications contributing to the patterns have been more elusive [3]. The primary focus of this study was to elucidate differences in presentations, treatments, and outcomes between men and women suffering from OOHCA and the accompanying contributing factors. It must be emphasized that outcomes between genders in the pre-hospital setting vary considerably from those occurring in-hospital and, therefore, any conclusions drawn from this study are not necessarily applicable to other healthcare settings.

Presentations between the genders

In our data set, women were, on average, eight years older than their male counterparts when experiencing OOHCA, a statistically significant finding that is consistent with many similar studies [12]. This trend may also be related to the differences in overall life expectancy between males and females: females living nearly five years longer than males [13]. It has been postulated that differences in age upon death between the genders are due to the disparity in estrogen and cortisol levels. Numerous studies have demonstrated the positive effects that estrogen has on cardiovascular health [12,14-15]. Intuitively, a drop in blood estrogen levels (as commonly seen in menopause) results in an increased incidence of cardiac-related issues, including cardiac arrest [14]. Intriguingly, estrogen has specifically been shown to decrease the cardiac arrest death pathway by anti-apoptotic mechanisms [15]. The underlying pathophysiology behind those mechanisms is the stabilization of the mitochondrial membrane and the prevention of the influx of calcium and potassium, which would normally lead to cell lysis [15]. Estrogen has also been shown to reduce injury-causing free radicals [15]. Given the beneficial role of estrogen in slowing the death cascade, some groups even propose a role for intravenous (IV) estrogen as a stabilizing drug in victims of cardiac arrest [15]. Additionally, cortisol has been shown to have deleterious effects on cardiovascular functionality and higher levels of cortisol have consistently been observed in males [16-17]. Irrespective of the underlying reason, the trend of women suffering from fewer episodes of cardiac arrest and at later ages remains consistent across many studies and medical literature [12,14-15].

Several cardiac rhythms can be observed during cardiac arrest: asystole, ventricular fibrillation (VFIB), ventricular tachycardia (VTACH), paced rhythm (PACED), and pulseless electrical activity (PEA). Past studies have concluded that patients initially presenting with VFIB or VTACH are more likely to have positive outcomes than those presenting with asystole or PEA [18]. Initial rhythm, therefore, has some predictive value regarding patient survivability. In our study, women were, to some extent, more likely to initially present in asystole and VTACH while men presented more frequently in PEA and VFIB. Numerous studies have revealed the disparity between men and women regarding arrhythmias although none have definitively concluded the underlying causes [3]. One study, however, suggested that women present more often with non-shockable rhythms due to having lower rates of ischemic heart disease [3]. Another study found that disparities in initial rhythms can have increased variability depending on the woman’s current menstrual cycle stage [19]. Perhaps linking hormonal levels with arrhythmias will lead to definitive treatment and implementation in their prevention and remediation.

Treatments between the genders

Miami Fire Department’s deliverance of care was moderately equal between the genders. Prior studies performed in other municipalities observed significant differences in treatments rendered based on gender [3]. Comparatively, the current study failed to find significant discrepancies in ETT success rate (Figure 3, p = 0.20), final airway management (Figure 4, p = 0.15), and dextrose administration (Figure 5, p = 0.08). However, significant differences were found in the rate of ETT attempts (Figure 3, p = 0.04), number of epinephrine doses administered (Table 2, p = 0.03), and number of defibrillations administered (Table 3, p = 0.02) between genders.

The study found a statistically significant difference in ETT attempts between males and females: males receiving more ETT attempts than females, 92% vs 87%, respectively (p = 0.04). One practical explanation is the fact that ETT success rates were lower in men as compared with women, 32% vs 38%, respectively (Figure 3). Perhaps the lower success rate necessitated more ETT attempts in men. The researchers did not conclude the reasons behind this; they simply illuminated the finding. The discrepancies in success rates in ETT placement may be due to anatomical differences between the genders. Men have larger vocal folds, leading to deeper voices [20]. A successful intubation requires the practitioner to insert the endotracheal tube through the patient’s vocal cords and into the trachea. Conceivably, the relatively larger vocal cords cause a slight impedance of the passage. Additionally, female vocal cords tend to be whiter, thereby making them easier to visualize when performing endotracheal intubations [20].

The remaining statistically significant findings included differences in treatment: average number of doses of epinephrine per encounter (M: 4.39, F: 3.99; p = 0.03) and average number of defibrillations per encounter (M: 3.26, F: 1.94; p = 0.02). The observed differences in care for males and females may be attributed to unconscious social norms and the care providers’ perception of patient viability. Because female patients are generally older and, therefore, may be seen as less viable, the treatment may sometimes be less aggressive. To drive this point home with a hypothetical example: a 90-year-old patient with an extensive medical history would typically receive less aggressive treatment than an otherwise healthy, 40-year-old, regardless of gender. While the ethics governing such practices are controversial, they are nonetheless ubiquitously done throughout many healthcare settings and municipalities. This study could not obtain data suggesting the reasons behind this disparity in treatment and so explanations are solely based on the clinical experiences of the authors of this study, in addition to interviews with some of the Firefighter-Paramedics of Miami.

Outcomes between the genders

A cardiac arrest encounter treated by the Miami Fire Department only has three possible conclusions for the patient: pronounced dead on scene, pronounced dead in the emergency department (receiving hospital), or ongoing resuscitation in the receiving emergency department. Miami’s protocols allow for the lead paramedic to terminate resuscitation efforts based on various factors, including patient down time, patient condition, and onset of rigor mortis. If it can be established that the patient had a long down time, meaning that they were in cardiac arrest for an extended period of time (usually more than 30 minutes) prior to any treatment, one can conclude that the possibility of survival is not high enough to constitute prolonged treatment. Under this mentality, the lead paramedic can terminate resuscitative efforts with the permission of the medical director (the physician overseeing EMS protocols and practices) [9]. A patient may be pronounced dead in the emergency department if the acting physician concludes that the patient’s chances of survival are too low to constitute prolonged treatment. Ongoing resuscitation occurs when the hospital staff is still administering resuscitative measures upon the EMS crew’s departure from the hospital. Many possibilities constitute ongoing resuscitation including (but not limited to) the patient’s achievement of ROSC or the patient being deemed viable by the responding physician. Generally, ongoing resuscitation and ROSC are considered to be the best outcomes in a prehospital cardiac arrest encounter. Our data observed a larger percentage of women with the end status of ongoing resuscitation and similarly higher ROSC rates than men. Overall, this may suggest that women fare marginally better in OOHCA than men; however, it is worth noting that no significant difference was found in our subject population.

Our data demonstrated there were essentially no differences in ultimate prehospital outcome between the genders (Figure 6, p = 0.27) but there was an interesting difference in overall ROSC rates: 37% of women achieved ROSC as compared with 30% of men (Table 4). However, this result was not statistically significant (p = 0.08).

The precipitating condition or initial rhythm dictates the deliverance of care based on ALS algorithms. If the assumption is made that algorithms are followed, then it can be postulated that the difference in outcome between genders can be attributed to differences in biology. Although males received significantly more doses of epinephrine than females (Table 2, p = 0.03), the higher overall higher ROSC rate in women receiving IV epinephrine could be a result of physiological differences. Studies have shown that women may metabolize antiarrhythmic drugs differently than men, and the dissimilarities can become exaggerated at distinct points in the menstrual cycle, leading to inconsistent responses in treatment [19]. Other studies have corroborated that variations in pharmacobiology make women more responsive to IV therapy and resuscitative efforts [14].

By shedding light on gender differences in cardiac arrest, this study can bring awareness to any prejudices or other underlying causes of discrepancy in care that may exist regarding medical treatment. Innumerable possibilities may account for the current, observed differences. Perhaps women are viewed as less resilient or a man’s life is held in higher regard. Perhaps EMS personnel are unaware that the deliverance of care is unequal to begin with.

The discrepancies in care between genders observed in this study may be attributed to the interplay of multiple factors, including, but not limited to, physiological, biological, and social differences. However, the focus and implications of this study are not to determine the reasons behind the inequality but rather to expose it. Once exposed, the medical community and education programs can address the gender disparities in the treatment and outcomes of OOHCA.

Limitations

Our analyses were limited to the city of Miami, Florida, and the Miami Fire-Rescue Department. While gender distribution in the United States is relatively uniform, population demographics, city layout, and training of EMS personnel can vary greatly across municipalities; making the obtained results less generalizable. Miami’s racial demographics differ significantly from the United States average: a higher percentage of Hispanics (71.2% in Miami, vs 17.8% nationally) and a higher percentage of African-Americans (18.8% vs 13.3% nationally) are two of the more notable differences [10,21]. The racial makeup of a city is an important consideration because arrest outcomes can significantly vary among different races and socioeconomic statuses [2]. Our dataset also has a lower percentage of patients presenting with an initial shockable rhythm (12% in Miami vs. 24% nationally) [22]. This is a pertinent characteristic because cardiac arrest patients that initially present with a shockable rhythm tend to have higher rates of favorable outcomes [6]. All of Miami’s responding ambulances provide the advanced life support (ALS) level of care. Some systems, including Miami Fire-Rescue, use an all-ALS system, while others use a tiered response of ALS and a basic life support (BLS) response or solely a BLS system. With this in mind, it is difficult to generalize our findings to other EMS systems because of the potential disparity in training and capabilities [23].

The retrospective nature of our study precludes the determination of causality and allows for a selection bias to influence our results. The researchers also did not control for any confounders, such as patient age, which could appreciably impact the aggressiveness of treatment. Generally, a much older patient is regarded as less viable and may not always receive the same amount of cardiac arrest interventions. This is a pertinent confounder given the significant age difference between the men and women noted in this study (63.97 years for men vs. 71.81 for women).

The data was only based on the self-reported accounts of cardiac arrest episodes by the responding paramedics. All reports containing obvious reporting errors were eliminated from our data set. However, reports were all written in the uncontrolled environment of the prehospital setting and were only written and edited by the lead paramedic, so it is possible that many unnoticed reporting errors were not taken into account.

Post-hospital data, such as neurological capabilities and other end-organ damage, were not available. The number of patients who went on to survive for a prolonged period of time following ROSC also could not be obtained, preventing one from drawing comprehensive conclusions regarding true survivability. The researchers did, however, have access to the Utstein Survivability Report, which showed the number of patients that went on to be discharged, alive, from the hospital. Determining which particular patients were the ones who survived was not possible so the analysis of that report was limited.

A patient outcome for positive ROSC was deemed positive based on the achievement of ROSC alone, regardless of how brief the ROSC lasted. Other positive patient parameters, such as end-tidal CO_2_ and neurological functioning were not utilized in this study, further limiting generalizability regarding patient outcomes. We also were limited by incomplete data on bystander cardiopulmonary resuscitation (CPR), patient ethnicity, patient downtime, and patient medical history, and these were, therefore, not reported in our dataset.

Although we observed several differences in administered interventions between men and women, many of them did not satisfy statistical significance perhaps due to the relatively small sample size. The most compelling finding exemplifying this limitation was the deliverance of dextrose. Roughly 94% of applicable males received IV dextrose versus 77% of females. The large difference in percentages did not satisfy statistical significance due to the relatively small sample size. Future studies exploring this discrepancy should contain more patients in order to draw more definitive conclusions.

## Conclusions

When comparing males and females, subject demographics, presentations, treatments, and outcomes varied considerably. The largest disparities were observed in demographics and interventions. Of significance, women were older than men while men appeared to receive more aggressive care, significantly more doses of epinephrine, and a higher number of ETT attempts. Other non-significant observed trends in treatment included lower male ETT success rate and higher administration of dextrose to males. However, a trend of overall ROSC and viable transfer to the ED were higher in women than in men, despite receiving less chemical and electrical interventions.

Future studies are needed to determine causality in discrepancies between genders in addition to investigating differences in treatment in other areas of the United States. Results derived from the city of Miami may not reflect trends and practices seen in other cities. By identifying differences in treatment and outcomes, medical education and training can be curtailed accordingly to provide more effective care in the field.

## References

[1] (2018). AHA cardiac arrest statistics. Cardiac Arrest Statistics.

[2] Weisfeldt M, Becker L (2017). Racial differences in in-hospital cardiac arrest: good news: cautious optimism is welcome. JAMA Cardiol.

[3] Kim LK, Looser P, Swaminathan RV (2016). Sex-based disparities in incidence, treatment, and outcomes of cardiac arrest in the united states, 2003-2012. J Am Heart Assoc.

[4] Sanghavi P, Jena AB, Newhouse JP, Zaslavsky AM (2015). Outcomes of basic versus advanced life support for out-of-hospital medical emergencies. Ann Intern Med.

[5] Regitz-Zagrosek V (2012). Sex and gender differences in health: science & society series on sex and science. EMBO Rep.

[6] Bosson N, Kaji AH, Fang A, Thomas JL, French WJ, Shavelle D, Niemann JT (2016). Sex differences in survival from out-of-hospital cardiac arrest in the era of regionalized systems and advanced post-resuscitation care. J Am Heart Assoc.

[7] (2018). Division of fire-rescue. FIRE-RESCUE.

[8] (2018). National emergency medical services education standards paramedic instructional guidelines. https://www.ems.gov/pdf/education/National-EMS-Education-Standards-and-Instructional-Guidelines/Paramedic_Instructional_Guidelines.pdf.

[9] Keroff FM, Schrank KM, Adams PD (2017). Common EMS Protocols. Miami Beach Fire Dept.

[10] Bureau USC. Miami city, Florida Florida (2018). U.S. census bureau quickfacts: Miami City, FL. https://www.census.gov/quickfacts/fact/table/miamicityflorida.

[11] Grunau B, Reynolds J, Scheuermeyer F (2016). Relationship between time-to-ROSC and survival in out-of-hospital cardiac arrest ECPR Candidates: when is the best time to consider transport to hospital?. Prehosp Emerg Care.

[12] Mendelsohn ME (2002). Protective effects of estrogen on the cardiovascular system. Am J Cardiol.

[13] (2018). Life expectancy in the USA hits a record high. USA Today.

[14] Safdar B, Stolz U, Stiell IG (2014). Differential survival for men and women from out-of-hospital cardiac arrest varies by age: results from the OPALS study. Acad Emerg Med.

[15] Wigginton JG, Pepe Pe Fau, Idris AH (2010). Rationale for routine and immediate administration of intravenous estrogen for all critically ill and injured patients. Crit Care Med.

[16] Whitworth JA, Williamson PM, Mangos G (2005). Cardiovascular consequences of cortisol excess. Vasc Health Risk Manag.

[17] Verma R, Balhara YPS, Gupta CS (2011). Gender differences in stress response: role of developmental and biological determinants. Ind Psychiatry J.

[18] Bunch TJ, Hammill SC, White RD (2005). Outcomes after ventricular fibrillation out-of-hospital cardiac arrest: expanding the chain of survival. Mayo Clin Proc.

[19] Kim C, Fahrenbruch CE, Cobb LA, Eisenberg MS (2001). Out-of-hospital cardiac arrest in men and women. Circulation.

[20] Titze I (1994). Principles of Voice Production.

[21] (2018). U.S. census bureau quickfacts: United States. https://www.census.gov/quickfacts/fact/table/US/PST045216.

[22] Daya MR, Schmicker RH, Zive DM (2015). Out-of-hospital cardiac arrest survival improving over time: results from the resuscitation outcomes consortium (ROC). Resuscitation.

[23] National Academies of Sciences, Engineering Engineering, and Medicine (2017). Exploring Strategies to Improve Cardiac Arrest Survival: Proceedings of a Workshop. https://www.nap.edu/read/23695/chapter/1.

